# Effects of freezing rate on structural changes in l-lactate dehydrogenase during the freezing process

**DOI:** 10.1038/s41598-021-93127-6

**Published:** 2021-07-01

**Authors:** Haena Park, Jun-Young Park, Kyung-Min Park, Pahn-Shick Chang

**Affiliations:** 1 Department of Agricultural Biotechnology, Seoul National University, Seoul, 08826 Republic of Korea; 2 Department of Food Science and Biotechnology, Wonkwang University, Iksan, 54538 Republic of Korea; 3 Center for Food and Bioconvergence, Seoul National University, Seoul, 08826 Republic of Korea; 4 Research Institute of Agriculture and Life Sciences, Seoul National University, Seoul, 08826 Republic of Korea; 5 Center for Agricultural Microorganism and Enzyme, Seoul National University, Seoul, 08826 Republic of Korea

**Keywords:** Proteins, Biomaterials - proteins, Enzymes

## Abstract

Freezing is a common method for improving enzyme storage stability. During the freezing process, the freezing rate is an important parameter that can affect protein stability. However, there is limited information on the denaturation mechanisms and protein conformational changes associated with the freezing rate. In this study, the effects of freezing rate on activity loss and conformational changes in a model enzyme, l-lactate dehydrogenase, were evaluated. Enzyme solutions were frozen at various rates, from 0.2 to 70.6 °C/min, and ice seeding was conducted to reduce supercooling. The results demonstrated that fast freezing results in activity loss, structural changes, and aggregation. The residual activities at freezing rates of 0.2, 12.8, and 70.6 °C/min were 77.6 ± 0.9%, 64.1 ± 0.4%, and 44.8 ± 2.0%, respectively. As the freezing rate increased, the degree of dissociation and unfolding increased significantly, as determined using blue native-polyacrylamide gel electrophoresis and fluorescence spectroscopy. Moreover, a large number of amyloid aggregates were detected in samples frozen at a fast freezing rate (70.6 °C/min). The enzyme inactivation mechanism induced by fast freezing was proposed in terms of increased dehydration at the enzyme surface and an ice/unfroze solution interface, which could be helpful to establish a common understanding of enzyme inactivation during the freezing process.

## Introduction

Freezing is a common method used to facilitate the storage stability of enzymes in research and industry^1^. In a frozen state, the rates of chemical reactions (e.g., oxidation, hydrolysis, deamidation, etc.) and physical degradation reactions (e.g., aggregation, precipitation, etc.) decelerate drastically due to decreased molecular mobility in the solution at low temperature and high viscosity^2^. Therefore, most enzymes preserved in a frozen state can maintain their initial activity and structure over long-term storage^1,3^. However, although freezing can improve protein stability, several enzymes, for example, l-lactate dehydrogenase (LDH), catalase, and β-galactosidase, are susceptible to freezing and may lose activity during the process^4–6^. Various physicochemical changes occur during freezing that influence protein stability^7^. Once water is crystallized into ice, water molecules are eliminated from the liquid phase, decreasing protein-bound water molecules^1^. Moreover, as the ice crystals grow, the concentration of the solute rapidly increases, leading to the formation of an interface between the ice and unfrozen solution^8,9^. These changes could affect the conformational stability of proteins^10^.

There has been increased interest in the rational design of the freezing process to minimize activity loss^11^. Until now, the process for optimizing freezing or freeze-drying of enzyme is still empirical and nonsystematic approach based on trial and error, controlling freezing conditions (e.g., type of buffer salts, surfactants, saccharides, and etc.)^3^. The empirical approach collecting data from multiple trials will be time-consuming and no guarantees of product quality. On the other hand, a systematic approach that is based on well-accepted scientific principles would increase production efficiency and achieve consistent high quality of products. However, there are still limitations to conduct the rational design of the freezing process, because the enzyme deactivation mechanism during the freezing process is not fully elucidated. Therefore, a study of individual stress factors can aid the design of a freezing process that minimizes stress and ensures the desired final product quality. During the freezing process, the freezing rate is one of the critical conditions determining enzyme stability, and a correlation between enzyme activity and freezing rate has been reported by several researchers^4,12^. However, few studies and industrial cases have considered and controlled the freezing rate during the freezing process. Furthermore, few studies have analyzed the effect of freezing rate on enzyme structure. Determining the degree of structural change according to the freezing rate is critical because protein structure is closely linked to stability. Furthermore, the enzyme inactivation mechanism during freezing can be suggested based on the results of the analysis of structural changes. The in-depth understanding of the inactivation mechanism is eventually required for a rational designing of the freezing process.

Therefore, in the present study, the effects of freezing rate on enzyme stability during the freezing process were evaluated by measuring activity loss and conformational changes. LDH (EC 1.1.1.27) was used as the model enzyme because it is a freeze-labile protein that loses its activity after freezing^4,8,13^. Three different freezing methods and rates were used: freezing in a deep freezer (− 75 °C), liquid-mediated freezing (LMF), and freezing in liquid nitrogen. The LMF system, based on freezing the sample in a container filled with isopropyl alcohol (IPA), was designed to easily and finely control the freezing rate and heat transfer rate by modulating solvent temperature. Conformational changes in LDH according to the freezing rate and degree of dissociation and unfolding were determined using blue native-polyacrylamide gel electrophoresis (BN-PAGE) and fluorescence spectroscopy. Dynamic light scattering (DLS) and fluorescence microscopy were used to analyze the size distribution and morphology of the denatured LDH after freezing.

## Materials and methods

### Materials

LDH (type II, from rabbit muscle, suspended in 3.2 M (NH_4_)_2_SO_4_, 830 units/mg protein), β-nicotinamide adenine dinucleotide (NADH) in a reduced form, and Tris base were purchased from Sigma-Aldrich (St. Louis, MO, USA). Pyruvate of 97.0% purity was obtained from Kanto Chemical Co. (Tokyo, Japan). IPA of 99.5% purity was purchased from Samchun Chemical Co. (Seoul, Republic of Korea). All other chemicals were of analytical grade.

### l-Lactate dehydrogenase assay

The LDH enzyme assay was performed at 25 °C using a slight modification of Bagchi's method^14^. The reaction mixture consisted of 11.4 mL of 0.2 M Tris–HCl buffer (pH 7.4), 0.4 mL of 6.6 mM NADH, 0.4 mL of 30 mM pyruvate, and 0.4 mL of 1.8 μg/mL LDH solution. The reaction was initiated by the addition of the enzyme solution. LDH activity was determined as the reduced absorbance of NADH at 340 nm using a spectrophotometer (UV-2450, Shimadzu, Kyoto, Japan). One unit of LDH activity was defined as the amount of enzyme that converted 1 μmol of NADH into 1 μmol of NAD per minute under assay conditions. Protein concentration was determined spectrophotometrically at 280 and 260 nm using the following Eq. (1):

1 $${\text{Protein}}\;{\text{concentration}}\;({\text{mg/mL}}){\text{ }} = {\text{ }}(1.55{\text{ }} \times {\text{ A}}_{{280}} ) - (0.76{\text{ }} \times {\text{ A}}_{{260}} )$$

where A_280_ and A_260_ are the absorbance values at wavelengths of 280 and 260 nm, respectively.

### Freezing process

**Figure 1:**
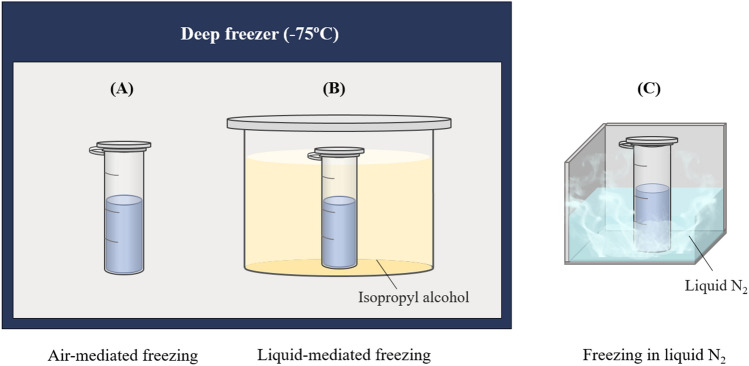
Schematic diagrams of the three different freezing methods. (**A**) Air-mediated freezing, (**B**) liquid-mediated freezing, and (**C**) freezing in liquid nitrogen (N_2_).

The LDH was dissolved in 0.2 M Tris–HCl buffer (pH 7.4) at a final concentration of 20 μg/mL (16.6 units/mL). The composition of the LDH solution can affect protein stability during freezing^4^. The total sample volume was 1 mL, and samples were frozen in 2 mL polypropylene tubes. Enzyme samples were frozen at different freezing rates using three different methods: air-mediated freezing, the LMF system, and freezing in liquid nitrogen (Fig. 1). For each method, ice seeding was performed before freezing. Ice nucleation was controlled at a specific temperature to ensure reproducibility of the freezing rate^7,15^. In the first step of ice seeding, enzyme samples were cooled to − 1 °C (the ice nucleation temperature) in a chilling block (Cole-Parmer Benchtop, Vernon Hills, IL, USA) and held for 2 min. In the second step, a tweezer, pre-cooled in liquid nitrogen for 35 s, was briefly dipped into the sample.

All samples were frozen until they reached the final temperature (− 50 °C), followed by thawing in a 25 °C water bath for 4 min at a thawing rate of 14 °C/min. The LDH assay was performed twice to quantify activity loss, before and after freezing. LDH structural analysis was carried out on samples freeze-thawed five times and used to detect subtle changes. The temperature of the enzyme solution during freezing was monitored using a thermometer (MTM-380SD, Lutron, Taipei, Taiwan). A thermocouple was mounted between the surface and bottom of the enzyme solution. Temperature changes were recorded using a data logger attached to the thermometer. The freezing rate was calculated as the average of post-nucleation cooling rates in the temperature range of 0 to − 5 °C, known as the zone of maximum ice crystal formation^16^.

### The liquid-mediated freezing system

The LMF system was designed to control the freezing rate of the enzyme solutions, with each enzyme solution frozen in a container filled with pre-cooled IPA (Fig. 1). The freezing rate was determined using the temperature difference between the solution and the IPA. The temperature difference was caused by the pre-cooled IPA temperature, which was modulated by the cooling time of the solution in a deep freezer (− 75 °C). IPA (400 mL) was added to each IPA container, a polypropylene cylinder. IPA temperature was measured as described in the section above.

### Blue native-polyacrylamide gel electrophoresis

BN-PAGE was performed using 0.002% (w/v) Coomassie Blue G-250 for the cathode buffer (pH 7.0), 3.3% polyacrylamide for the stacking gel (pH 7.0), and 6.5% polyacrylamide for the resolving gel (pH 7.0). Electrophoresis was conducted at 90 V and 4 °C for 4 h. After electrophoresis, the gel was incubated in a fixing solution (40% [v/v] ethanol, 10% [v/v] acetic acid) for 1 h and washed in distilled water for 9 h. The gel was then sensitized in 0.02% (w/v) sodium thiosulfate for 1 min. Silver staining was conducted at 4 °C using 0.1% (w/v) silver nitrate solution with a gentle rocking motion. The gel was developed in a solution consisting of 3% (w/v) sodium carbonate and 0.05% (v/v) formaldehyde. The formaldehyde was added just before use. Development was terminated by adding 5% (v/v) acetic acid solution.

### Fluorescence spectroscopy

Fluorescence spectroscopy was performed with a fluorescence spectrometer (FluoroMate FS-2, Scinco Co., Seoul, Republic of Korea). Intrinsic fluorescence emission spectra of freeze-thawed 1 mL enzyme solutions (16.6 units/mL) were recorded at 25 °C in a 10 mm optical path length quartz cuvette (Hellma Analytics, Müllheim, Germany). The excitation wavelength was 280 nm, and emission spectra were collected from 300–600 nm in 0.1-nm increments. The slit width for excitation and emission was 5 nm, and the photomultiplier tube voltage was set to 400 V.

### Fluorescence microscopy

In 100 μL volumes (16.6 units/mL), freeze-thawed enzyme solution was mixed and stained with 1 μL of 100 μM Nile red solution in ethanol before microscopic examination. Observations were made under a fluorescence microscope (DE/Axio Imager A1 microscope, Carl Zeiss, Oberkochen, Germany) equipped with a Zeiss filter cube no. 15 (EX BP 546/12, BS FT 580, EM LP 590) and a 100 × Plan-Neofluar objective lens. Images were acquired with an AxioCam HRC CCD camera (Carl Zeiss) and processed using AxioVision software (Ver. 4.8, Carl Zeiss).

### Dynamic light scattering

The size distribution of enzyme aggregates was analyzed via DLS using a Zetasizer Nano ZS90 instrument (Malvern Instruments Ltd., Worcestershire, UK). Scattered light intensity was monitored at 90°, and hydrodynamic sizes were calculated using the autocorrelation function of the Zetasizer Nano software. Freeze-thawed 1 mL enzyme solutions (16.6 units/mL) were added to disposable cuvettes (Ratiolab, Dreieich, Germany) with a path length of 10 mm. The solvent refractive index was set to 1.33, the viscosity of dispersant was 0.8872 cP, and the dispersant dielectric constant was 78.5. All measurements were carried out at 4 °C to suppress additional aggregation due to heat.

## Results and discussion

### Effect of freezing rate on the activity of l-lactate dehydrogenase

**Figure 2:**
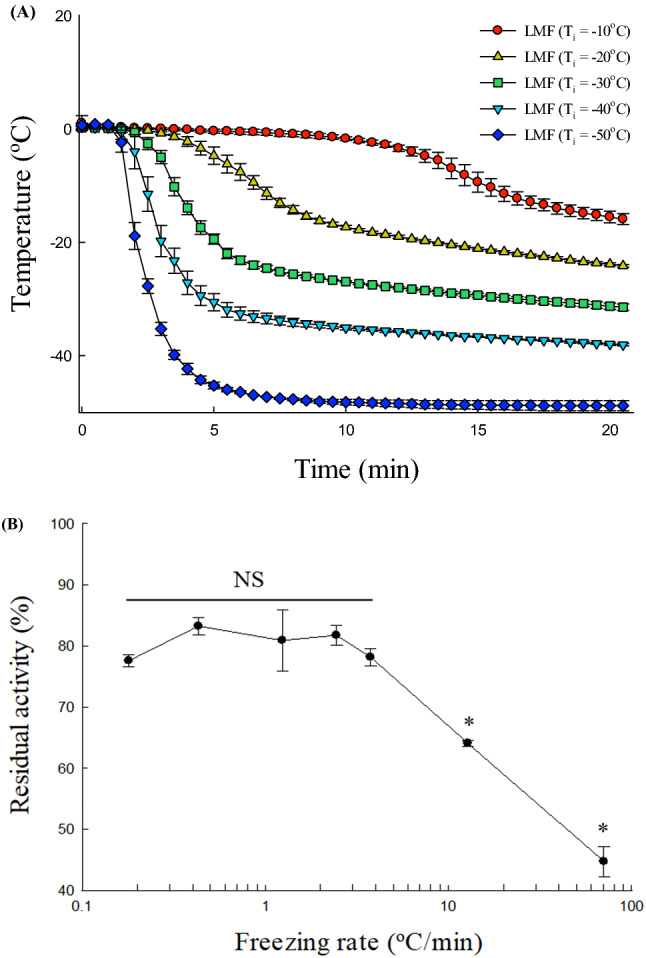
(**A**) Freezing curves of the l-lactate dehydrogenase (LDH) solution (20 μg/mL, 0.2 M Tris–HCl buffer, pH 7.4) at different liquid-mediated freezing (LMF) conditions [T_i_] =  − 10, − 20, − 30, − 40, or − 50 °C). T_i_, the temperature of isopropyl alcohol just before freezing LDH samples. (**B**) Effect of freezing rate on the activity of LDH dissolved in 0.2 M Tris–HCl buffer (pH 7.4). Results are expressed as the means ± SDs. Asterisks (*) indicate significant differences (*p* < 0.05). *NS* non-significant difference.

The LMF system was devised to freeze the enzyme solution at various freezing rates to determine the effect of freezing rate on enzyme stability during the freezing process. This system controls the sample freezing rate by changing the initial temperature (T_i_) of the solvent (IPA). The temperature profiles of the enzyme solution monitored at each T_i_ showed that the freezing rate increased as T_i_ decreased (Fig. 2A). In all freezing conditions, the temperature decreased continually even in the region of phase change because of freezing point depression of the enzyme solution (0.2 M Tris–HCl) with a high concentration of solutes.

Freezing rate can be expressed by a variety of definitions including time–temperature methods, the velocity of ice front, appearance of a frozen sample, and thermal methods^17^. The time–temperature method, temperature change per unit time or time to transverse a given range of temperatures, is the most frequently used when the ice crystal structure influences the quality of a frozen product. The freezing rate was calculated in the temperature range of 0 to − 5 °C (Table 1), known as the zone of maximum ice crystal formation at which nucleation and ice crystal growth occur. Because, the zone of the maximum is a critical region to determine the freezing rate, as the ice crystal size is determined by the time taken for the phase change^18^. The freezing rate of each method increased, as expected, in the following order: air-mediated freezing (0.2 °C/min) < LMF (0.4–12.8 °C/min) < freezing in liquid nitrogen (70.6 °C/min). Notably, supercooling did not occur during the freezing process due to ice seeding (Fig. 2A). The ice nucleation temperature in all freezing treatment groups was controlled at a specific temperature (− 1 °C). Enzyme solutions frozen using an identical method can have a different size distribution of ice crystals from vial to vial because ice nucleation is a stochastic process^1^. Therefore, precisely controlling ice nucleation was necessary to investigate the interfacial area effect, which is affected by ice crystal size, on enzyme stability during the freezing process. Supercooling was not triggered, indicating that ice nucleation was well controlled in all treatment groups.

**Table 1:** Residual activities of l-lactate dehydrogenase according to freezing rate.

Freezing methods			Freezing rate ( °C/min)	Residual activity (%)
Air-mediated freezing			0.2 ± 0.01	77.6 ± 0.9^a^
LMF	T_i_ (°C)	− 10	0.4 ± 0.04	83.3 ± 1.2^a^
		− 20	1.2 ± 0.19	80.9 ± 4.1^a^
		− 30	2.5 ± 0.41	81.8 ± 1.4^a^
		− 40	3.8 ± 2.06	78.2 ± 1.2^a^
		− 50	12.8 ± 3.30	64.1 ± 0.4^b^
Freezing in liquid nitrogen			70.6 ± 8.09	44.8 ± 2.0^c^

*LMF* liquid-mediated freezing, *T*_*i*_ initial temperature of isopropyl alcohol.

^a–c^Different superscript letters denote significant differences between treatments (*p* < 0.05). The means were compared using ANOVA and ranked using Duncan’s multiple range test.

In the thawing process, the ice recrystallization is also an additional damage factor to proteins^4^. The water molecules on the small ice crystal surface having high free energy are melted and migrate to the large ice crystal surface, resulting in ice crystal growth to decrease free energy (i.e., ice recrystallization)^18^. Thawing slowly generally causes ice recrystallization to be more significant, causing more protein denaturation (Supplementary Fig. S1). Therefore, LDH activity was assayed after fast thawing (14 °C/min) to minimize the effect of recrystallization. As shown in Fig. 2B, the residual activity of LDH decreased drastically as the freezing rate increased. When the freezing rate was relatively slow (0.18–3.76 °C/min), the highest residual activity levels were observed (80.3 ± 2.2%). At the intermediate freezing rate (12.8 °C/min), the residual activity was 64.1 ± 0.4%, decreasing to 44.8 ± 2.0% at the fastest freezing rate of 70.6 °C/min; thus, more than half of the initial activity was lost. These results indicate that enzyme activity loss can be minimized at relatively low freezing rates.

**Figure 3:**
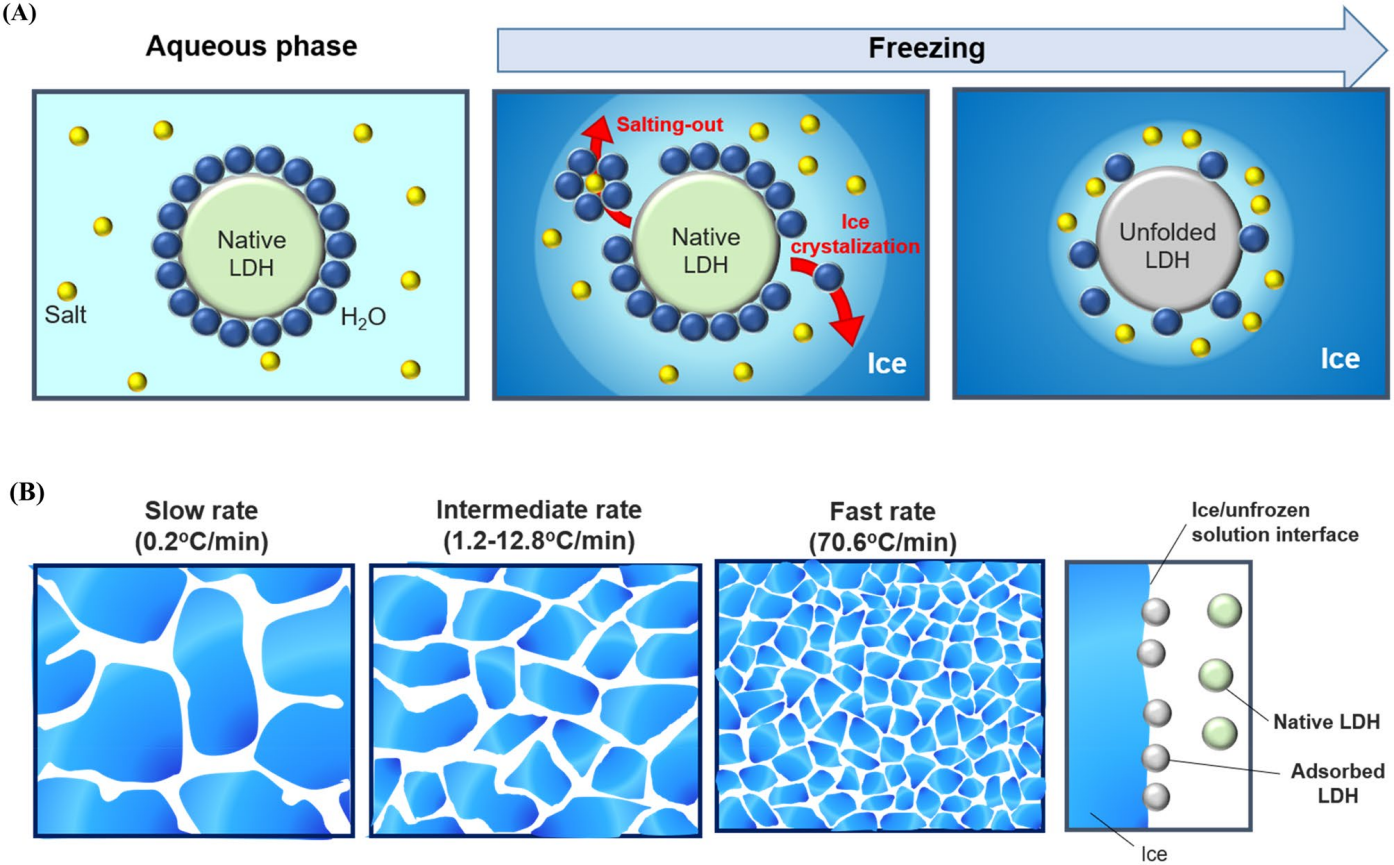
Schematic illustration of the mechanisms of enzyme destabilization by fast freezing. (**A**) Increased dehydration at the enzyme surface. (**B**) Formation of an ice/unfrozen solution interface.

As indicated, loss of enzyme activity during the freezing process could be caused by ice crystallization (Fig. 3). In the aqueous phase, the interaction between proteins and water molecules plays an important role in protein structural stability^10^. However, once the water is crystallized into ice, the protein surface is dehydrated as water molecules around the protein are removed, which can destabilize protein structure^19^. Moreover, as the ice crystals grow, the solute concentration increases drastically^20^. Under a freeze-concentrated state, the interaction between the solute and water molecules strengthens, and the number of water molecules interacting with proteins is reduced. Consequently, fast freezing rates lead to fast dehydration rates, causing severe protein damage. The drastic decrease in various interactions between water molecules and proteins, which contribute to protein structural stability, can affect protein conformation. Alternatively, previous research implicated the ice/unfrozen solution interface as a major contributor to protein damage during freezing^5^. When proteins are adsorbed onto the ice/unfrozen solution interface, they can lose their original structure, which leads to activity loss^21^. The results of this study support this assumption because fast freezing results in a large interfacial area and greater damage, leading to overall enzyme activity loss. Consequently, these results strengthen the above two hypotheses.

### Dissociation and unfolding of l-lactate dehydrogenase after fast freezing

**Figure 4:**
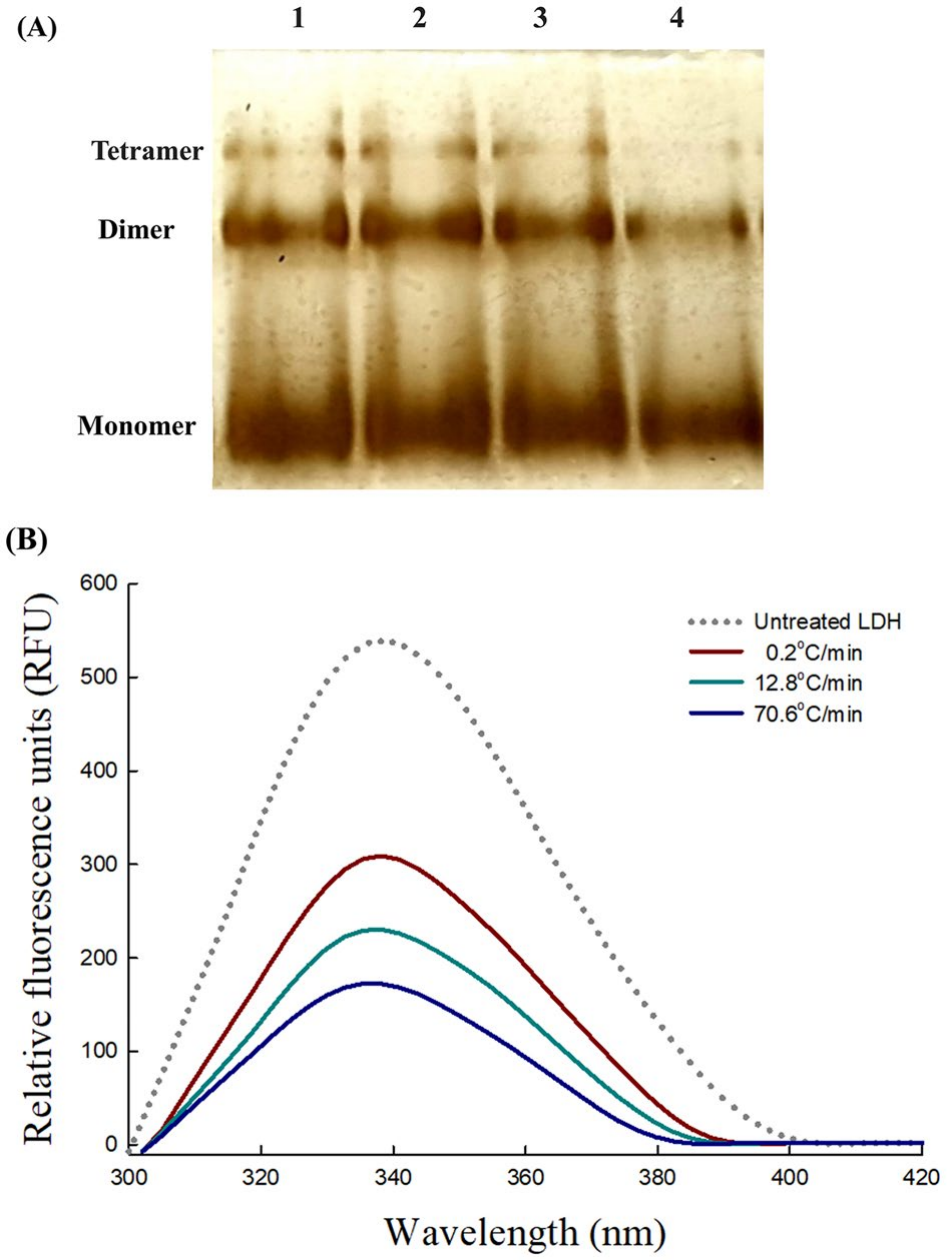
(**A**) Blue native-polyacrylamide gel electrophoresis profile of LDH after silver staining following the freeze–thaw process repeated five times. The concentration of the LDH solution was 40 μg/mL. Lane 1 was for untreated LDH, Lanes 2, 3, and 4 were for freeze-thawed LDH treated at freezing rates of 0.2, 12.8, and 70.6 °C/min, respectively. The full-sized gel is presented in the supplemental information (Supplementary Fig. S2). (**B**) Intrinsic fluorescence emission spectra (300–420 nm) of 20 μg/mL LDH in 0.2 M Tris–HCl buffer (pH 7.4) after the freeze–thaw process was repeated five times. LDH solution was frozen at a freezing rate of 0.2, 12.8, or 70.6 ºC/min. The frozen samples were thawed in a water bath at 25 ºC for 5 min before analysis.

Figure 2B illustrates that the freezing rate is a significant determinant of final enzyme activity after freezing. It was also expected that the freezing rate would change the protein structure based on enzyme structure–function associations. For LDH, a tetrameric enzyme, subunit dissociation represents a critical structural change as it causes loss of activity. The BN-PAGE gel image of LDH after freezing at different rates (0.2, 12.8, and 70.6 °C/min) is presented in Fig. 4A. In this gel system, three bands were observed in each lane, distributed according to size^22^. The intensities of the bands assigned to tetramers and dimers were significantly weaker at the fastest freezing rate (70.6 °C/min) compared with the low and moderate freezing rates (0.2 and 12.8 °C/min). These results indicate that a faster freezing rate promotes protein dissociation. Three bands were also detected in the untreated sample lane, but those were caused by the Coomassie Blue G250 used in the cathode buffer^23^. The dye has a mild negative charge, essential for the migration of proteins, but causes some dissociation of proteins^22^. Therefore, freezing induces quaternary structural changes, and an increased freezing rate accelerates denaturation. The loss of the LDH quaternary structure was one of the major structural changes that occurred during fast freezing.

When multimeric proteins are dissociated, they can easily unfold because their structural stability is lower than that of intact proteins. The fluorescence signal of tryptophan in proteins is sensitive to their surroundings and changes according to protein folding status^24^, making fluorescence spectroscopy suitable for detecting subtle conformational changes induced by fast freezing. There was a significant decrease in fluorescence emission intensity at 340 nm as the freezing rate increased (Fig. 4B). LDH frozen at 70.6 °C/min exhibited the largest decrease in fluorescence emission intensity, to 68.2 ± 3.0% compared with the native state (i.e., untreated sample). The results suggest that fast freezing also causes tertiary structural changes in the enzyme. These conformational changes were confirmed by the maximum emission wavelength (E_max_) shift of the tryptophan residues, which depends on the chemical environment^25^. The E_max_ exhibited a blue shift at the freezing rate of 70.6 °C/min (from 339–335 nm). For LDH, which contains tryptophan residues on its surface in the folded state, a blue shift can occur when tertiary conformational changes lead to the burying of tryptophan residues in the protein core^26^. Also, a significant drop in the intrinsic fluorescence intensity coupled with the blue shift can be described as aggregation. When tryptophans are accessible to quenchers such as histidines, phenylalanines, and disulfide bonds upon aggregation, the tryptophan fluorescence intensity decrease^27^. Consequently, the fast freezing caused quaternary and tertiary structural changes and aggregate formation. The degree of conformational change increased at the fast freezing rate, emphasizing the effect of freezing-induced damage in proteins.

### The effect of freezing rate on the aggregation of l-lactate dehydrogenase

**Figure 5:**
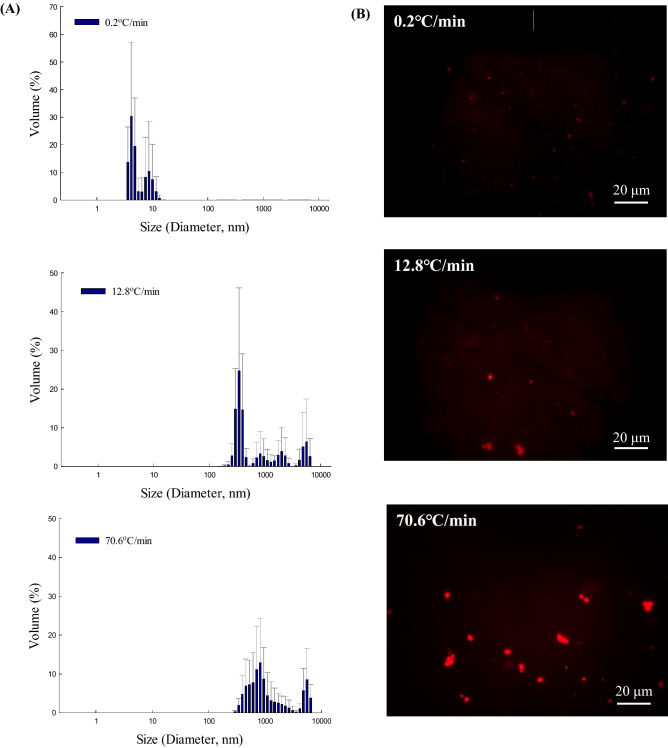
(**A**) Dynamic light scattering analysis of 120 μg/mL LDH in 0.2 M Tris–HCl buffer (pH 7.4) after the freeze–thaw process was repeated five times. The freezing rates were 0.2, 12.8, and 70.6 °C/min. (**B**) Nile Red fluorescence micrographs of 20 μg/mL LDH in 0.2 M Tris–HCl (pH 7.4) after the freeze–thaw process was repeated five times. LDH solution was frozen at a freezing rate of 0.2, 12.8, or 70.6ºC/min. The frozen samples were thawed in a water bath at 25 ºC for 5 min before analysis.

Aggregate formation represents a quality defect in protein products because it decreases total activity, and aggregates can become nuclei for the growth of larger clusters. Thus, it is important to analyze the conditions that trigger aggregate formation. A blue shift in fluorescence spectroscopy can be attributed to protein aggregation due to buried tryptophan residues accumulating in the protein. As the enzyme loses its quaternary and tertiary structure after freezing, low conformational stability increases the possibility of aggregate formation. DLS analysis was carried out to measure the particle size distribution in freeze-thawed enzyme samples and determine the effect of freezing rate on aggregate formation. DLS is suitable for analyzing colloidal systems such as protein solutions^28^; however, in a multimodal distribution, the size distribution as expressed by light scattering intensity was skewed to large particles because of their high sensitivity^29^. Thus, the size distribution of protein aggregates should be expressed as volumes converted from intensity readings to reduce the emphasis on large particles. In contrast to untreated LDH (0.6 nm diameter, data not shown), large particles were detected in all freezing-treated groups (Fig. 5A). At the lowest freezing rate (0.2 °C/min), most particles had a diameter of approximately 10 nm. At higher freezing rates (12.8 and 70.6 °C/min), the proportion of particles with a size of approximately 1000 nm increased. The proportion of particles larger than 400 nm accounted for 42.2% of total particles at 12.8 °C/min and 92.9% at 70.6 °C/min. Larger, aggregated particles were formed as the freezing rate increased.

Freeze-thawed samples were stained with Nile red and visualized using fluorescence microscopy to confirm the formation of large particles. Nile red emits strong fluorescence upon binding to hydrophobic surfaces of proteins exposed during conformational changes^30^. Therefore, Nile red staining allowed more effective visualization and detection of LDH protein aggregates in the fluorescence photomicrographs. Red dots indicating aggregates were not observed, as expected, in untreated LDH samples (data not shown). By contrast, red dots were detected in freeze-treated LDH samples (Fig. 5B), demonstrating that aggregation was induced by freezing. These microscopic images also showed that the fluorescence emission intensity and red particle size increased with the freezing rate, consistent with the DLS results. It was concluded that fast freezing caused severe aggregation, which results from structural changes. In general, non-biological particles such as nanoparticles are prone to aggregation during slow freezing which allows the significant exclusion of nanoparticles from the ice crystal, resulting in aggregation^31^. On the contrary, LDH, which is a biological particle, is vice versa, because denatured proteins tend to oligomerize by various interactions between amino acid residues. Therefore, fast freezing of LDH caused structural changes and following aggregation, corresponding to these explanations. The formation of aggregates gives insight into the enzyme deactivation mechanism during the fast freezing process. The multimeric LDH lost its quaternary structure during the freezing process, and the dissociated subunits easily unfolded because of their low structural stability. They could then be re-assembled based on hydrophobic interactions. In the case of fast-frozen LDH, subunits dissociated from LDH formed a greater amount of aggregates.

**Figure 6:**
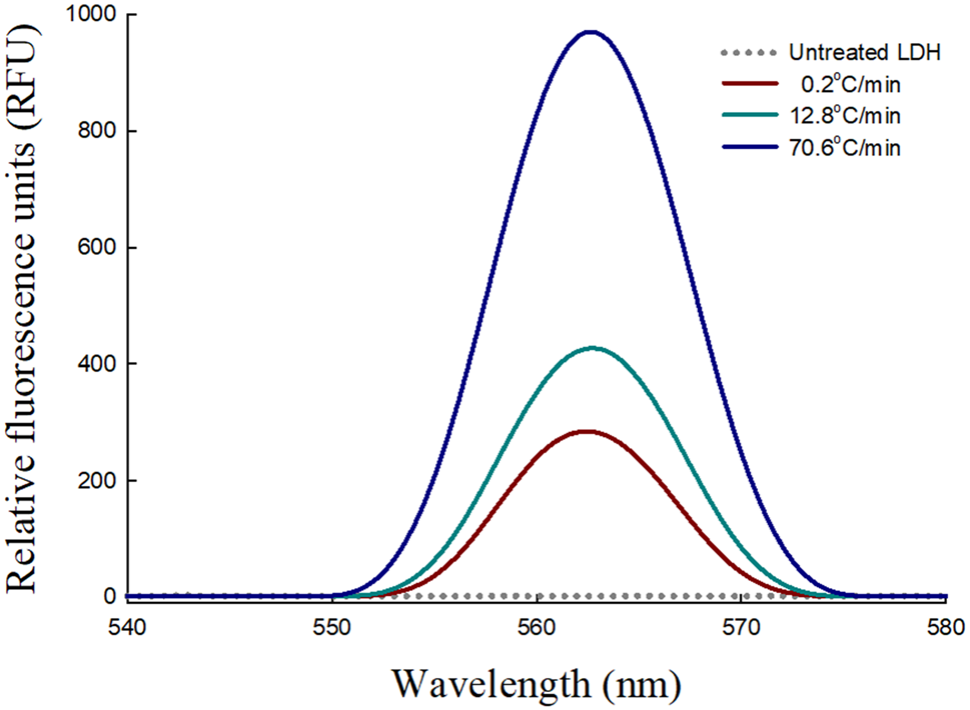
Intrinsic fluorescence emission spectra (540–580 nm) of 20 μg/mL LDH in 0.2 M Tris–HCl buffer (pH 7.4) after the freeze–thaw process was repeated five times. LDH solution was frozen at a freezing rate of 0.2, 12.8, or 70.6º C/min. The frozen samples were thawed in a water bath at 25 ºC for 5 min before analysis.

Interestingly, in the fluorescence spectroscopy analysis, unique elements in the fluorescence emission spectra of freeze-thawed LDH solution were detected at approximately 564 nm (Fig. 6). A significant increase in intensity was observed as the freezing rate increased. The intrinsic fluorescence of proteins stems from aromatic residues; however, recent research has identified a novel intrinsic protein fluorescence pattern in the visible range, despite excitation in the UV range^32^. This unique emission pattern is probably due to amyloid aggregates, which have a high proportion of β-sheets^33^. It is suggested that extensive arrays of hydrogen bonds in β-sheet structures give rise to electron delocalization, which shifts fluorescence emissions into the visible range^34^. Thus, the intrinsic fluorescence can be an indicator of amyloid formation, which is supported by recent reports^17,35,36^. The extent of amyloid formation after the freezing process and fluorescence intensity exhibited a positive correlation; hence, these results demonstrated that the fast freezing of LDH caused the amyloid aggregation.

## Conclusions

This study investigated the effects of freezing rate on a model enzyme’s activity and structure after the freezing process. The decreased enzyme activity after fast freezing demonstrated that LDH was severely destabilized as the freezing rate increased. Analysis of conformational changes and the freezing rate showed that the degree of dissociation and unfolding increased during fast freezing compared with slow freezing. The unfolded proteins aggregated, mostly as amyloids. This phenomenon would be associated with the increased rate of dehydration at the protein surface and the adsorption of proteins onto the ice/unfrozen solution interface. Consequently, this study is an important attempt to understand the enzyme inactivation mechanism during fast freezing. Further researches on various freeze-labile enzymes would be required to establish a common understanding of freezing enzymes for a rational designing of the freezing process, which would increase production efficiency and achieve consistent high quality of products in industry.

## Supplementary Information

Supplementary Figures.
