# Supplementary material for: Effects of freezing rate on structural changes in l-lactate dehydrogenase during the freezing process
Source: Sci Rep. 2021 Jul 1;11:13643. doi: 10.1038/s41598-021-93127-6 (PMC8249661; doi:10.1038/s41598-021-93127-6)
Supplement: Supplementary file 1 — Supplementary Figures. [file 41598_2021_93127_MOESM1_ESM.docx]

Supplemental Information

**Effects of freezing rate on structural changes in L-lactate dehydrogenase during the freezing process**

Haena Park^1^, Jun-Young Park^1^, Kyung-Min Park^2,5^, and Pahn-Shick Chang^1,3,4,5*^

**Affiliations**

^1^*Department of Agricultural Biotechnology, Seoul National University, Seoul 08826, Republic of Korea*

*^2^Department of Food Science and* *Biotechnology, Wonkwang University, Iksan, 54538, Republic of Korea*

^3^*Center for Food and Bioconvergence, Seoul National University, Seoul 08826, Republic of Korea*

^4^*Research Institute of Agriculture and Life Sciences, Seoul National University, Seoul 08826, Republic of Korea*

*^5^Center for Agricultural Microorganism and Enzyme, Seoul National University, Seoul 08826, Republic of Korea*

Supplementary Data


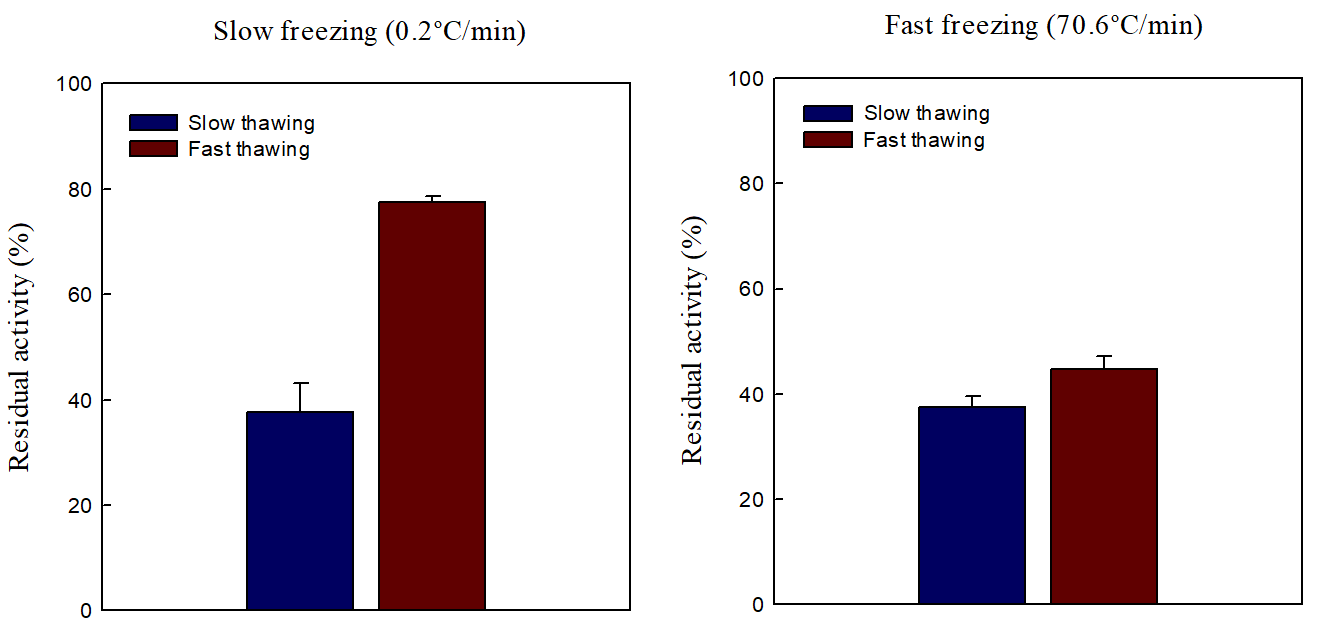
(A) (B)

**Fig. S1.** Effects of thawing rate on the residual activity of L-lactate dehydrogenase after freezing at different conditions. (A) Slow freezing rate (0.2℃/min). (B) Fast freezing rate (70.6℃/min).

**
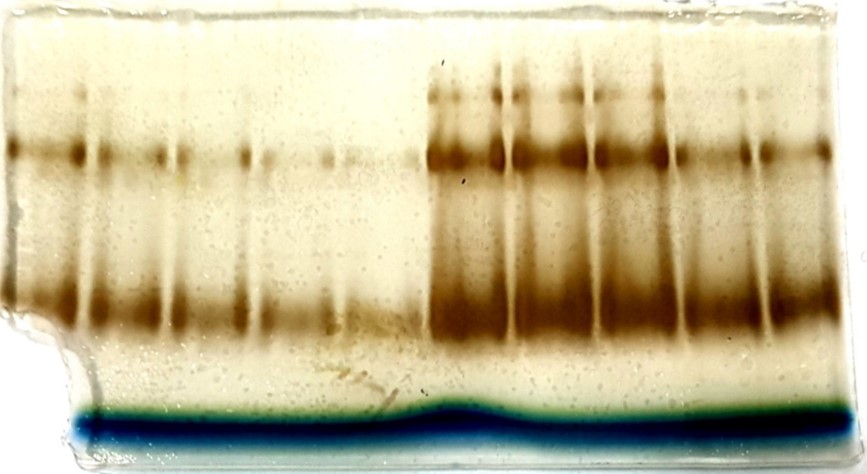
**

**Fig. S2.** Full-length gel of Fig. 4. (A), indicating a cropped section by the red-dotted lines.
